# The Value of Educational Messages Embedded in a Community-Based Approach to Combat Dengue Fever: A Systematic Review and Meta Regression Analysis

**DOI:** 10.1371/journal.pntd.0001278

**Published:** 2011-08-23

**Authors:** Nada Al-Muhandis, Paul R. Hunter

**Affiliations:** School of Medicine, Health Policy and Practice, University of East Anglia, Norwich, United Kingdom; Centers for Disease Control and Prevention, Puerto Rico, United States of America

## Abstract

**Background:**

The effects of various dengue control measures have been investigated in previous studies. The aim of this review was to investigate the relative effectiveness (RE) of different educational messages embedded in a community-based approach on the incidence of *Aedes aegypti* larvae using entomological measures as outcomes.

**Methods and Findings:**

A systematic electronic search using Medline, Embase, Web of Science and the Cochrane Library was carried out to March 2010. Previous systematic reviews were also assessed. Data concerning interventions, outcomes, effect size and study design were extracted. Basic meta-analyses were done for pooled effect size, heterogeneity and publication bias using Comprehensive Meta-analysis. Further analysis of heterogeneitity was done by multi-level modelling using MLwiN. 21 publications with 22 separate studies were included in this review. Meta-analysis of these 22 pooled studies showed an RE of 0.25 (95% CI 0.17–0.37), but with substantial heterogeneity (Cochran's Q = 1254, df = 21, p = <0.001,). Further analysis of this heterogeneity showed that over 60% of between study variance could be explained by just two variables; whether or not studies used historic or contemporary controls and time from intervention to assessment. When analyses were restricted to those studies using contemporary control, there was a polynomial relationship between effectiveness and time to assessment. Whether or not chemicals or other control measures were used did not appear have any effect on intervention effectiveness.

**Conclusion:**

The results suggest that such measures do appear to be effective at reducing entomological indices. However, those studies that use historical controls almost certainly overestimate the value of interventions. There is evidence that interventions are most effective some 18 to 24 months after the intervention but then subsequently decline.

## Author Summary

Dengue fever is a mosquito-borne viral infection that is widespread in the tropics. Each year there are an estimated 50 million infections worldwide. Preventing infection relies on controlling the mosquitoes that spread disease. Unfortunately it is still not clear what does and does not work in the control of the mosquito vector. There have been several systematic reviews into control of dengue fever but still no consensus has been reached. This lack of consensus reflects the substantial heterogeneity in published effectiveness of studies of dengue control interventions. Prior systematic reviews have not adequately addressed this heterogeneity. We used multi-level modelling meta regression to investigate what variables modify the effectiveness of studies of educational messages embedded in a community-based approach. Most of the between study variation was explained by two variables, study design and time from intervention to assessment. In particular, studies using historic controls substantially overestimated the effectiveness of the intervention compared to those studies using contemporary controls. When the analysis was restricted to just those studies using contemporary controls, effectiveness was highest about 12 to 24 months after intervention but then declined.

## Introduction

Dengue fever (DF) is an acute viral disease affecting all age groups. It occurs mainly in tropical and subtropical areas, the predominant vectors being the mosquitoes *Aedes aegypti* and *albopictus*, which become infected with any of the four dengue viruses and transmit the disease via a bite to humans [1]. Some 2.5 billion people (two-fifths of the world's population) are now at risk from dengue, and the WHO currently estimates that there may be 50 million dengue infections worldwide every year [2]. Depending on the year, tens to hundreds of thousands of cases of the severe and potentially fatal form of the disease, dengue haemorrhagic fever, and dengue shock syndrome (DHF/DSS) occur [3].The incidence of DF has increased dramatically in recent decades. Its proliferation is influenced by many mechanisms – these include population growth with unplanned urbanisation (and consequent overburdening of water and sanitation systems), increases in domestic and international travel, transportation of commodities such as tyres, lack of political will to intervene, and limited financial and human resources to implement effective control measures [4]. The disease has become endemic to more than one hundred countries in Africa, the Americas, the Eastern Mediterranean, South-East Asia and the Western Pacific. Of these, South-East Asia and the Western Pacific are the most seriously affected. There is currently no vaccination for DF, and no medications that can treat DHF or DSS directly, so at present the only way of controlling or preventing the spread of the virus is to combat vector mosquitoes directly.

For many years, spraying with insecticides, such as malathion, has been the main method of control, though this has often had limited success [5]. Other interventions aimed at controlling the mosquito population, have been tested with varying success. For example, the Puerto Rican government, in response to the threat of a DHF epidemic, developed an integrated approach consisting of community-based dengue control programs to complement traditional chemical-based approaches [6]. They encouraged the public to reduce or eliminate containers in and around homes, gardens and villages. These containers, which include discarded plastic packaging, metal cans, and rubber car tyres, are capable of holding water which would then harbour larvae, and allow mosquitoes to proliferate [7]. It has become clear, from the number of projects that have been initiated in recent years that community-based programs are now regarded by both national and international health agencies as the primary long-term solution for prevention and control of DHF/DSS in Asia and the Americas [3].

A recent systematic review carried out by Erlanger and colleagues investigated the effect of different types of dengue vector control interventions, including biological, chemical, environmental and integrated vector management, on well established entomological parameters [4]. Their aim was to compare the effects of these interventions, in order to find the most efficacious. They identified 56 publications with extractable data that had compared the impact of 61 different dengue control interventions with control communities or with the same community prior to the intervention. The authors concluded that dengue interventions are effective in reducing vector populations, particularly when interventions use a community-based integrated approach. An earlier systematic review by Heintze *et al*. specifically looked at community-based dengue control programmes, and concluded that the evidence that such programmes were effective, either alone or in combination with other programmes was weak [7]. Yet another recent systematic review also concluded that there was little evidence to support the efficacy of mosquito abatement programs due to poor study design and lack of congruent entomological indices [8] An important criticism that can be levelled at these systematic reviews is that there was substantial heterogeneity in study design and in the size of any effect that made it difficult to draw definitive conclusions. In particular some studies used historical control periods whilst others used other contemporary communities as controls. Many of the studies included multiple interventions in combination whilst others studies were of a single intervention. Furthermore the control communities may or may not have had one of more interventions themselves. We argue that these issues make it particularly difficult to disentangle the value of educational messages embedded in a community-based approach, or identify the most successful approach. Although Erlanger and colleagues did undertake subgroup analysis around types of intervention, neither of these studies adequately investigated sources of heterogeneity in effect size (the magnitude of any association between the outcome and predictor) making the drawing of any definitive conclusions problematic.

One of the recent trends in meta-analysis has been the increasing use of methods that aim to investigate causes of heterogeneity in effect size between published studies rather than rely on pooled effects sizes that can often be difficult to interpret [9], [10]. By this way it is hoped that additional insights can be gleaned into how study design and context, such as use of control interventions in control groups, may affect the outcome. Such insights could give some understanding of in what situations these interventions may, or may not, have benefit. This paper reports a deeper analysis of papers that have attempted to determine the impact of educational messages embedded in a community-based approach, which we define as community based intervention that had any element where members of the public were given information or exhortations intended to change their behaviour, on entomological indicators of risk of dengue disease.

## Methods

The primary outcome of this review was to establish which, if any, of these interventions was most effective in reducing larval indices. Usually entomological effectiveness was measured using one or more of three widely utilised indices: the Breteau index (BI), container index (CI) and house index (HI). The BI specifies the number of containers with *Aedes* spp. larvae per 100 houses, the CI represents the percentage of water containers positive for *Aedes* spp. larvae, and the HI gives the percentage of houses with water containers holding immature *Aedes* spp. [4]. In addition, one study used the average number of positive containers per house (C+/H) [11].

### Inclusion criteria

Included studies were required to: firstly refer to control of dengue fever, and secondly have studies investigating an educational intervention alongside a ‘control’ approach or standard management program. Studies also had to look at quantitative outcomes, whether these were the BI, CI, HI or C+/H. Next, these studies had to be community-based, whereby members of the community partook in the interventions or played a major role. Conversely, studies based in laboratory or semi-field settings were excluded, as were purely observational cross-sectional and qualitative studies. Studies were not limited by language of publication.

The primary measure of effect size was relative effectiveness (RE) with 95% confidence intervals. RE is the ratio between the entomological index in the intervention group and in the control group. Consequently the more effective the intervention the lower the RE. An RE of 1.0 would indicate no effect. Where confidence intervals were not given, these were back-calculated from the *P* value. Where only the entomological index/indices were presented for each group RE and its 95% confidence intervals were estimated using Monte Carlo modelling with @Risk™. The distributions of the indices for the intervention and control groups were taken from the papers. Then values were repeatedly sampled from each distribution and the value sampled from the intervention distribution divided by that sampled from the control sample to give the RE. From the repeat samplings the distribution of the RE was then determined to give mean and 95% confidence intervals.

The review carried out by Erlanger *et al*. investigated a range of interventions, including entomological and community measures taken in a variety of settings [4]. The objective of this review was to systematically analyse only the publications which included an educational element to their interventions (even if other non-educational interventions were also included). However, we used a rather broad definition of educational intervention to include any community based intervention that had any element where members of the public were given information or exhortations intended to change their behaviour. This was followed by a rigorous up-to-date search strategy, detailed below, which was carried out in order to retrieve references which had been produced since publishing date of the existing review (September 2008).

### Search Strategy

A structured electronic search of Medline, EMBASE, Web of Science and the Cochrane Database of Systematic Reviews was carried out up to March 2010. This was performed in the format: [dengue or dengue haemorrhagic fever or dengue virus or *Aedes aegypti*] AND [arthropod vectors] AND [community based] AND [intervention]. Reference lists were checked for additional publications to the ones found in the initial search, which fulfilled the inclusion criteria.

From the initial search results, all titles and abstracts were assessed independently by two reviewers, with disagreements being resolved by discussion. From these, a list of papers to include was made, and full text articles obtained.

### Data Extraction

Once the publications had been assessed as meeting the prescribed quality and inclusion criteria, and having considered the references used by Erlanger *et al*., data was extracted systematically, using a standardised form. Data was extracted from the existing systematic review, but also updated with the most recent studies found in the search. Where follow-up occurred over several time points, the longest follow up time point results were included, as this provides the most realistic indicator of long-term effectiveness of the intervention. Data was extracted on the outcome measure, study design, time of follow-up after intervention, what other interventions were used and the nature of the educational component.

### Statistical Analysis

Where confidence intervals were not presented in the original paper, these were derived by a process of back-calculation from the presented *P* value. Initial analyses were done with Comprehensive Meta-analysis (CMA) Version 2.2.050 [12]. All four main entomological indices were included in the analyses. If more than one entomological index were reported in the same study, then a single outcome measure was calculated as the geometric mean of the different entomological index by CMA using the within program option to combine effect sizes from different types. CMA was used to calculate heterogeneity, determine potential effects of publication bias and pooled estimates of effect size. In order to determine whether combining REs using different entomological indices was valid, Pearson correlation coefficients were calculated as was paired t tests between them.

Subsequent analyses of the impact of moderator variables were done using MLwiN [13]. A basic three level model was constructed to account for studies with multiple comparisons [14]. Each of the putative modifier variables were put singly into the model and those with p<0.2 included in a multiple modifier model. In the multiple model, any modifier with p> = 0.2 was then removed and the model rerun until all modifiers in the model had p<0.2. The proportion of the between study variance explained by the final model was derived from τ^2^ (between-studies variance) in the model with no modifiers and in the final model.

## Results

**Figure 1:**
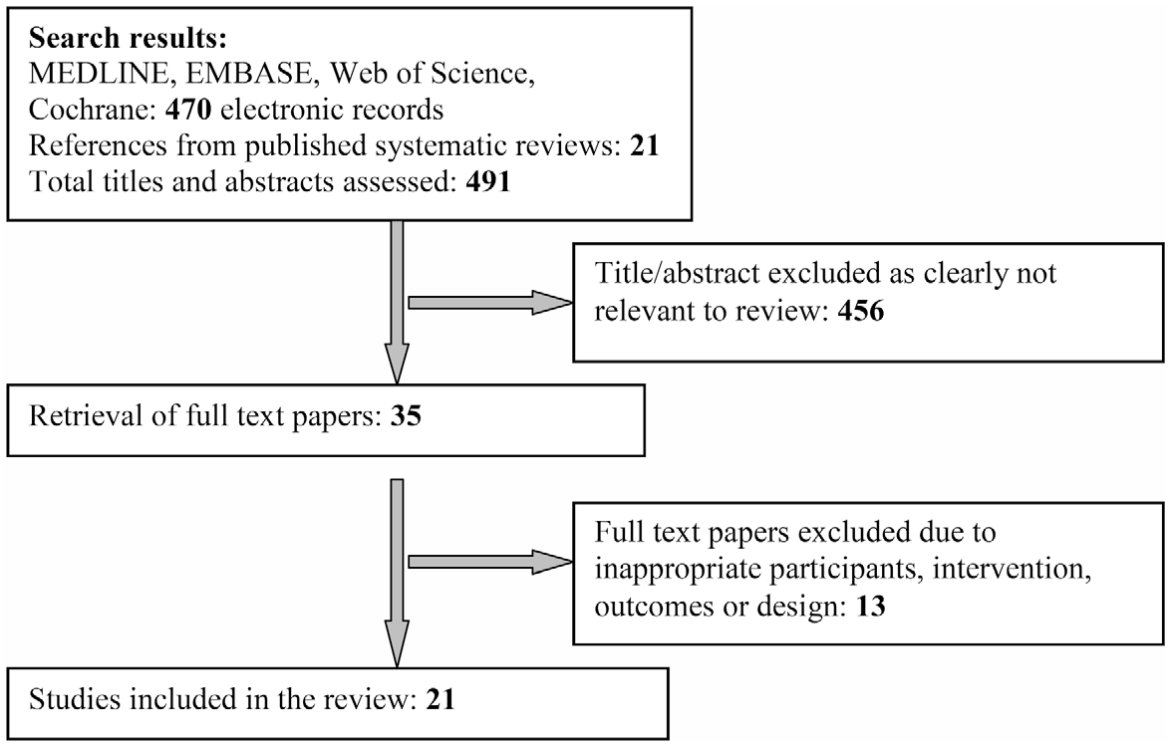
Study flow diagram of the search process and inclusion of studies into this review.

Searches of Medline, EMBASE, Web of Science and the Cochrane Database of Systematic Reviews identified 491 original papers for assessment. Figure 1 shows the flow diagram detailing the search process and inclusion of studies in this review. Of these 491 articles, 456 articles were excluded based on abstract alone because of inappropriateness of subject or study design. A total of 35 papers were obtained in full text. Of these, 14 full text papers were excluded, deemed to be unsuitable with regard to participants, the intervention used, outcomes of the study, or study design. This left 21 papers of which 11 were based in South America, 9 were based in South East Asia, and the remainder were based in Fiji and French Polynesia. The earliest study was published in 1967 [15] and the latest in 2009 [5]. One paper [11] had two study arms that included interventions of interest and each study arm is referred to as separate study were included, giving 22 studies in total.

Studies varied with regard to types of educational component, study design and control groups. The included studies and summary of their characteristics are listed in Table S1. The educational components included the use of print or broadcast media, public lectures, in-home training by public health staff, home visits and targeting school children. The exact mix of interventions varied between studies. Three different approaches were used in the study designs: 6 studies used an historical control period, measuring outcomes in the same village at baseline and at a later time point (‘historical’ control group), 11 studies included a control arm with no additional treatment as well as an intervention arm (‘no treatment’ control group), and 5 studies included a control arm exposed to some anti-mosquito activity, along with an intervention arm (‘some intervention’ control group). The studies also varied in terms of whether or not the intervention communities received other interventions. A total of 9 studies included some form of chemical intervention, as well as the educational component. This varied from the use of malathion spraying both in- and outdoors, to larviciding with the use of abate. Another 8 studies used various additional “other” (i.e. not chemical) measures in the intervention group – these ranged from covering and disposal of containers capable of holding water, to community clean-up campaigns, to the use of other species such as *Mesocyclops* in order to predate the *Aedes* spp.

**Figure 2:**
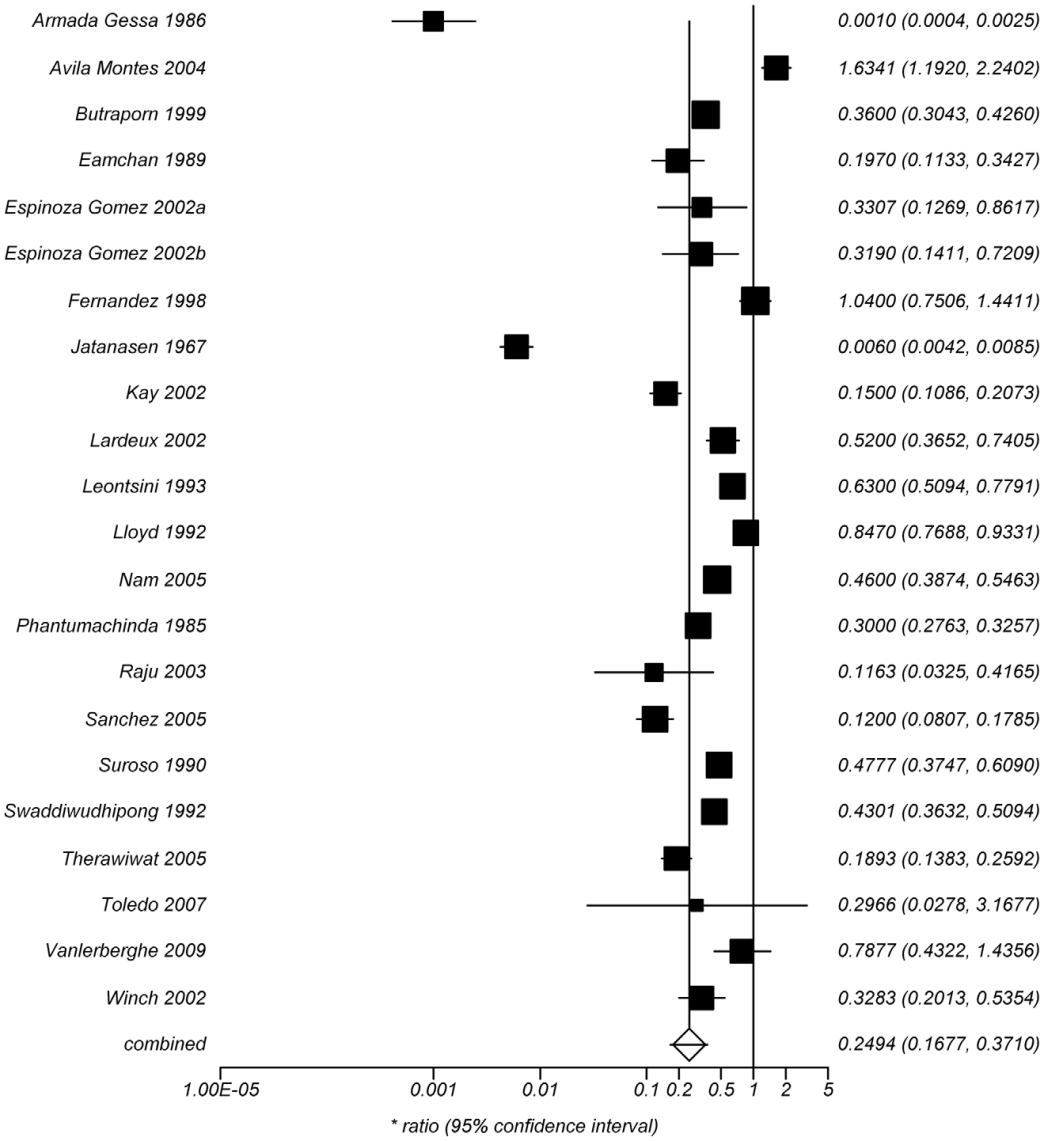
Performance of educational, chemical and other interventions, against dengue vector outcome measures. ^*^The diamond represents the combined relative effectiveness (RE); squares represent the RE of individual studies; the size of the square represent the weight given to the study in the meta-analysis; and the horizontal lines represent the 95% confidence limits. Risk Ratio equates to Relative Effectiveness, values of < 1 indicate lower entomological indices in the intervention compared to the control arm. The lower the value the more effective the intervention.

**Figure 3:**
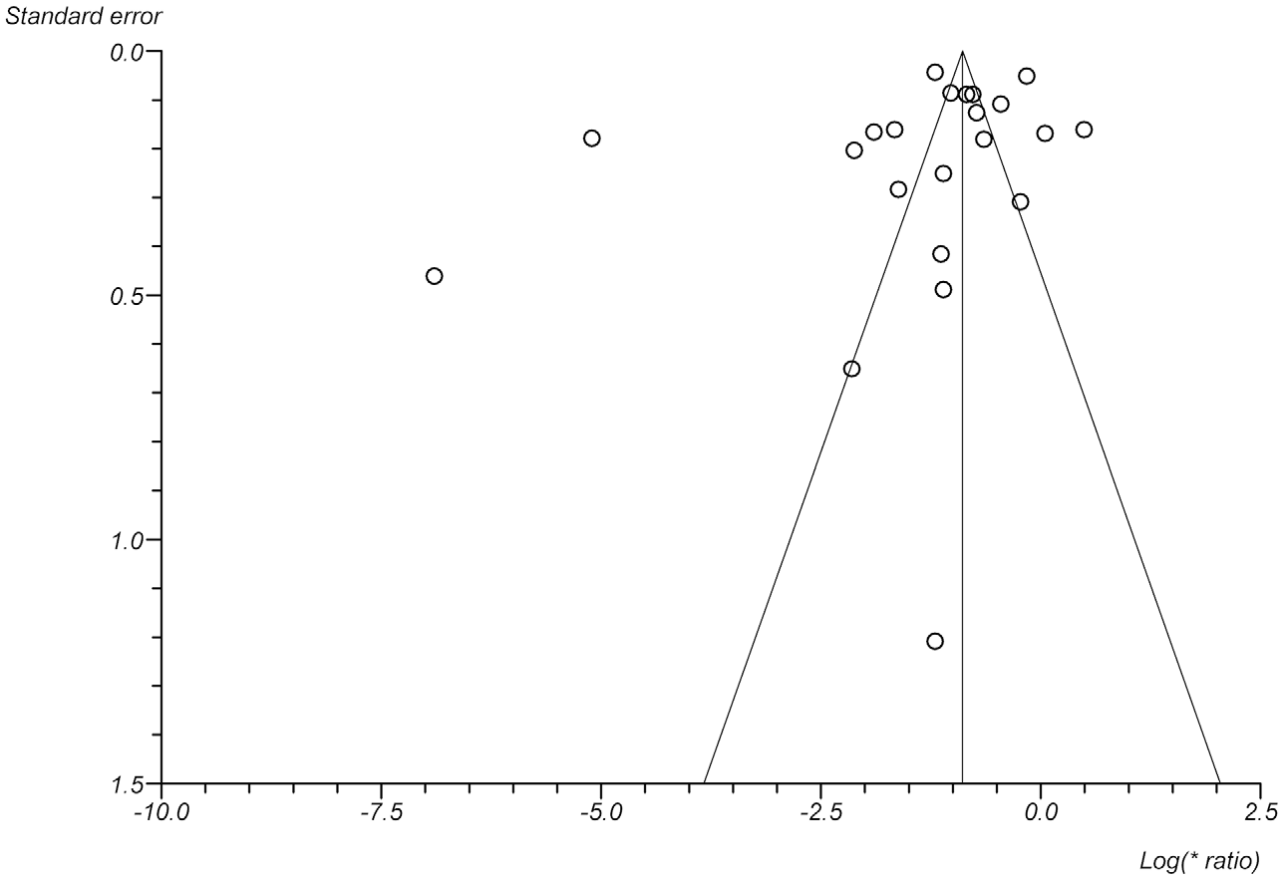
Funnel plot of standard error by log relative effectiveness.

The Pearson correlation coefficients for the REs from the three main entomological indices showed high correlation between them BI-CI 0.68, BI-HI 0.66, CI-HI 0.97. Furthermore, there was no significant difference in the mean RE given by the different entomological indices using a pared t test. We conclude that combining the different entomological indices was valid. The result of the meta-analysis performed on all 22 studies is shown in Figure 2. Using the random effects model, the pooled risk ratio was 0.25 (95% CI 0.17–0.37). However, there was substantial heterogeneity in the effect size (Cochran's Q = 1254, degrees of freedom (df) = 21, p = <0.001). There was no evidence of publication bias (Figure 3).

In order to investigate the sources of heterogeneity further a series of multi level meta-regression analyses were run with potential modifier variables. The results of the initial analyses are shown in table 1. The most significant single modifier variable was whether or not the study used a historical (comparing the same community before and after the intervention) or a contemporary control (comparing the intervention community with another control community). Those studies that used contemporary controls had a much reduced effect size compared to historical controls (Regression coefficient (B) = 2.08, Standard Error (SE) = 0.65). Two other predictor variables, combining a chemical with the community intervention and time at follow-up almost achieved significance. Whether or not other interventions were also used (but not including chemicals) did not achieve statistical significance. Two measures of time at follow-up were tested, the untransformed and log transformed months. The results were very similar between these two time measures and the untransformed used in subsequent analyses as this was marginally more significant and also easier to interpret. In addition the relationship between chemical spraying and RE was further tested as some studies included chemical spraying in both intervention and controls and others in intervention only. Perhaps not surprisingly, chemical spraying where this was applied in both intervention and control arms had almost no effect on RE whilst chemical spraying in the intervention arm but not control arm was associated with a significant improvement in RE (−2.08, SE 0.78).

**Table 1:** Single predictor variable analysis of relative effectiveness (RE)^a^.

Predictor variable		Log RE	SE	T	d.f.	P
Combined intervention	None	0				
	Chemical	−1.362	0.823	1.65	19	0.114
	Other	−0.220	0.834	1.59	19	0.795
Combined intervention with Chemical	No	0				
	Yes but chemical spraying also applied to controls	0.039	0.563	0.07	19	0.945
	Yes and chemical spraying not applied to controls	−2.079	0.775	2.68	19	0.015
Study design	Historical control	0				
	Contemporary control	2.076	0.648	3.20	20	0.0045
Any intervention in controls	No	0				
	Yes	0.645	0.823	0.78	20	0.442
Any intervention in controls, excluding historic controls	No	0				
	Yes	−0.045	0.368	0.12	14	0.904
Time at follow-up/m		−0.081	0.042	1.93	17	0.071
Log time at follow-up/m		−1.135	0.593	1.91	17	0.073

a Note that negative Log RE is associated with greater reduction in entomological indices in the intervention.

All variables with p<0.2 in the single modifier variable analyses were included (historic or contemporary controls, time at follow-up and chemical spraying in intervention but not control group) in a final model as shown in table 2. It can be seen that two modifier variables remain historical v contemporary control and study duration. In particular those studies using contemporary controls gave much smaller effect sizes than those using historical controls (B = 2.21, SE = 0.66) and effect sizes improved with longer delays till the follow-up assessments (B = −0.083/month, SE = 0.03). Using chemicals in the intervention group but not in controls was not significant. These three variables were able to explain 64% of the between study variance in the original dataset, though the remaining between study variance was still significant (τ^2^ = 1.07, SE = 0.39, z = 2.77, p = 0.006). Excluding chemical spraying from the model was still able to explain 61% of the between study variance.

**Table 2:** Final model showing impact of modifiers on relative effectiveness of intervention on entomological indices.^a^

		Log RE	SE	T	df	P
Time at follow-up/m		−0.083	0.030	2.77	15	0.014
Study design	Historical control	0				
	Contemporary control	2.209	0.655	3.37	15	0.004
Chemical spraying in intervention but not control	No	0				
	Yes	−0.774	0.651	1.19	15	0.253
Constant		−1.764	0.678	2.60	15	0.020

a Negative Log RE equates to increased effectiveness.

**Figure 4:**
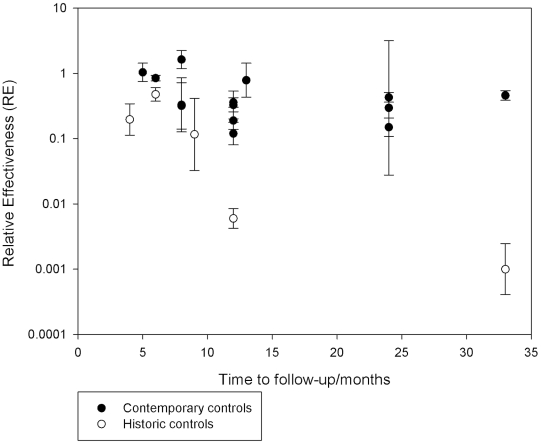
Midpoint of relative effectiveness of studies using by control and by time from intervention to assessment.* Graph only based on 19 studies as follow-up time not given for three studies.

The relationship between RE, choice of control and time to assessment is illustrated in figure 4. Here the difference between RE and choice of control is very clear. It can also be seen that within each category of control the relationship between RE and time to follow-up is more complex. For those studies with historic controls there is a very steep decline with time to assessment. For those studies with contemporary controls there is still a suggestion of a decline over the first 12 months then this levels out and possibly even reverses. This is reflected in the regression equation (table 3) where Log RE is predicted by the time to assessment and time to assessment squared. Indeed, the polynomial equation of time to follow-up was able to explain 44% of the between study variance in the studies with only contemporary controls.

**Table 3:** Final model of modifiers when restricted to those studies with a contemporary control group.^a^

	Log RE	SE	T	df	P
Time at follow-up/m	−0.259	0.096	2.698	11	0.021
Time at follow-up/m squared	0.00623	0.00255	2.443	11	0.033
Constant	1.107	0.717	1.543	11	0.151

a Negative Log RE equates to increased effectiveness.

Chemical spraying in the intervention but not control arms of the study were also included (B = 0.54, SE = 0.44, p = 0.254). Although this variable was not significant and subsequently dropped from the final model it is notable that the chemical spraying was if anything associated with reduced effectiveness of the community intervention.

## Discussion

The pooled results of the 22 studies in this meta-analysis suggest an important impact of educational messages embedded in a community-based approach on reducing larval indices. However, there was substantial heterogeneity in effect size between the different studies. This large heterogeneity in effect size reflects the very different study designs in the included studies. As discussed above the studies may or may not have included interventions additional to the community components, they may have used historic or contemporary controls, the controls may or may not have had some form of non-educational intervention. Consequently interpretation of the pooled effect size is difficult. However, the majority of the heterogeneity was explainable by just two variables, the choice of control and the time from intervention to assessment.

The impact of choice of control was particularly marked with studies using historical controls finding much stronger effect sizes than those using contemporary controls (table 2). After adjusting for the time to assessment, anyone basing their judgement of the effectiveness of educational interventions based on historical controls would over-estimate the value of educational interventions by more than 10 fold compared to studies that used contemporary controls (RE = 13.2, 95%CI 4.1–42.5).

If the impact of one aspect of the study design is so great, it begs the question which is the correct study design to use. It could be argued both ways. In favour of the use of historic controls is the argument that at least the populations being compared are geographically the same. The arguments in favour of the contemporary controls include the fact that entomological indices may change from one time to another for reasons totally unrelated to the intervention. Indeed it could be argued that as interventions are usually implemented when the risk of dengue fever is particularly high, it is very likely that entomological indices will improve substantially whatever the intervention. The problems with historical controls are well known to medical researchers [16], [17]. Indeed, the comment has been made that “most historical control groups are compromised for some reason” [18]. In studies with historic controls it has also been argued that the biases are worse the longer the time between the control and intervention. Our analysis would certainly support this suggestion for entomological control measures. Consequently we would argue that studies using historic controls be excluded from any assessment of the effectiveness of dengue vector control programmes.

The polynomial relationship between RE and time to assessment is interesting. From the regression model of only those studies with contemporary controls, the highest effectiveness is seen somewhere around 18 months after the educational intervention. This is consistent with the suggestion that people may need time to learn, but after that their effort and good intentions may slip without reinforcement.

As regards the value of non community interventions in addition to the community interventions, we found little evidence of any effect. In our single modifier analyses additional chemical application did appear of value when the control group did not receive chemical applications. However, in the model with control type and time to assessment, it was not significant. In the model including only studies with contemporary controls there was no evidence that chemical application provided any additional value over that achieved by education alone. However, there were only two studies that included chemical application in the intervention arm and not the control arm [19], [20]. One of these studies supplied sand abate to the villagers, and the other used water treatment with temephos and outdoor spraying from the ground with malathion. Clearly, one cannot draw any definitive conclusions based on two studies in a meta-regression analysis. However, in this regard the study of Espinoza-Gomez and colleauges deserves special mention [11]. In this, well conducted study the authors randomly allocated houses to one of four intervention groups: no intervention, indoor chemical spraying only, education only and combined education and chemical spraying. For our meta-analysis, initialy compared education with no intervention and then compared education plus chemical with chemical only. In their original analysis using a two way ANOVA, the authors found that only education was effective at reducing larval indices and that chemical spraying gave no benefit either alone or in combination with education. In addition, a recent systematic review of the value of the effectiveness of peridomestic space spray also found little benefit of chemical spraying [21]. Why peridomestic spraying has uncertain benefit is unclear.

With regard to comparison of educational interventions against one another, results showed that no single intervention modality (such as the use of print or broadcast media, lectures, training by public health staff, home visits or targeting school children) nor the number of different modalities used together was found to improve the RE significantly (data not shown). However, few if any studies were designed to compare different educational intervention modalities and so we would argue that this issue remains unanswered until specific studies are designed to address the relative effectiveness of different educational modalities.

Although meta-analyses of experimental studies such as randomised controlled trials are usually taken to provide high quality evidence of cause and effect, meta-regression analyses as presented here have the evidential status of observational studies. One of the other issues with meta-analyses of public health interventions is that often it is impossible to adequately blind the study participants or the study investigators. For example this has been raised as a major issue for studies of the effectiveness of household water treatment [10], [22]. Wood *et al*., in their research into evidence of bias associated with different study designs, found that lack of blinding could be associated with an apparent effect of about 30% for subjective outcomes [23]. For objective outcomes they found no evidence of such bias. Clearly the studies included in the analysis were not blinded. Whether or not the entomological indices are subjective or objective measures are open to debate. We would argue that these indices are semi-subjective and are potentially open to some form or bias due to lack of blinding of the assessors. However, even accounting for this the RE is much greater than could be explained purely by observer bias.

It has been underlined by Erlanger *et al*., that ‘relative effectiveness’ as numerical evidence of reduction of entomological measures, does not necessarily equate directly to reduction in pathogen transmission' [4]. Other factors such as villages sharing water supplies and garbage disposal may also enable disease transmission even if control within the village was good [11]. Gubler and Clark, in their review of dengue interventions as a whole, state that it must be kept in mind that in the types of community approaches assessed in this review, with these types of community approaches and strategies, it is expected that ordinary members of a community assume responsibility for activities that have historically been conducted by governmental bodies [3]. They suggest that for this reason, it would be very optimistic to expect immediate changes.

In their systematic review of the literature, Erlanger et al. concluded that dengue vector control is effective in reducing vector populations [4]. Since that review three additional systematic reviews have been published none of which came to the same conclusion as Erlanger [7], [8], [21]. These later studies basically came to the conclusion that the quality of the published evidence, something that Erlanger et al. did not adequately address, was too poor to form a definitive conclusion. We would generally agree with the three later studies. In particular we have shown that a major problem is that studies with historical controls strongly overestimate RE compared to those with contemporary controls. Nevertheless, after accounting for the use of historic controls we still found evidence that supports the value of educational messages embedded in a community-based approach in reducing entomological indices of risk. We also showed that there is some evidence that the value of such interventions may decline after 18 to 24 months. With the evidence currently available it is not possible to say what types of educational modalities are most effective. There is a need to reassess whether other interventions add any further value to educational interventions. Finally, the issue also remains whether entomological indices alone is always translated into disease reduction.

## Supporting Information

Table S1 **Characteristics of and references to included studies.**
(DOCX)

Checklist S1 **PRISMA 2009 checklist.**
(DOC)
