# Supplementary material for: The Value of Educational Messages Embedded in a Community-Based Approach to Combat Dengue Fever: A Systematic Review and Meta Regression Analysis
Source: PLoS Negl Trop Dis. 2011 Aug 23;5(8):e1278. doi: 10.1371/journal.pntd.0001278 (PMC3160295; doi:10.1371/journal.pntd.0001278)
Supplement: Table S1 — Characteristics of and references to included studies. (DOCX) [file pntd.0001278.s001.docx]

Table. Characteristics of and references to included studies.

| Study | Location | Time to follow up/ months | Outcome measure | Intervention arm: educational interventions | Intervention arm: non-educational interventions | Control type | Whether treatment given to control group^a^ |
| --- | --- | --- | --- | --- | --- | --- | --- |
| Armada Gessa^1^ | Cuba | 33 | HI | P, B, T | Chemical | Historical | Not applicable |
| Avila Montes^2^ | Honduras | 8 | BI, CI, HI | P, I, T, H, S | Caretaker to prevent infestation, group interviews | Contemporary | No treatment |
| Butraporn^3^ | Thailand | 12 | BI | P, I, T | Chemical | Contemporary | No treatment |
| Eamchan^4^ | Thailand | 4 | BI, CI, HI | I | Chemical | Historical | Not applicable |
| Espinoza Gomez^5^ | Mexico | 8 | C+/H | P, B, I, H | None | Contemporary | No treatment |
| Espinoza Gomez^5^ | Mexico | 8 | C+/H | P, B, I, H | Chemical | Contemporary | Some intervention |
| Fernandez^6^ | Honduras | 5 | CI | P, B, H | None | Contemporary | Some intervention |
| Jatanasen^7^ | Thailand | 12 | CI | P, B, H | Chemical | Historical | Not applicable |
| Kay^8^ | Vietnam | 24 | BI | P, B, I, T, H, S | Community clean-up campaign; Mesocyclops | Contemporary | No treatment |
| Lardeux^9^ | French Polynesia | Unknown | CI | P, B, I, H | Release of fish, sealing cisterns, polystyrene beads, chemical if these failed | Contemporary | No treatment |
| Leontsini^10^ | Honduras | Unknown | BI | P, I, T, H | Community clean-up, sewage maintenance | Contemporary | No treatment |
| Lloyd^11^ | Mexico | 6 | BI, CI, HI | P, I, H | None | Contemporary | No treatment |
| Nam^12^ | Vietnam | 33 | CI | P, B, I, T, H, S | Mesocyclops, container removal, salt into vases, community clean-ups | Contemporary | No treatment |
| Phantumachinda^13^  , | Thailand | Unknown | BI | P, I, T, S | Chemical | Historical | Not applicable |
| Raju^14^ | Fiji | 9 | BI, CI, HI | B, T, H | Elimination of breeding foci, protection of breeding water, trash disposal | Historical | Not applicable |
| Sanchez^15^ | Cuba | 12 | CI, HI | I, T, S | Eliminate containers, covering tanks, cleaning public/inhabited areas | Contemporary | Some intervention |
| Suroso^16^ | Indonesia | 6 | BI, CI, HI | P, B, H, S | Disposal/covering of containers/ tyres, clearing/covering reservoirs | Historical | Not applicable |
| Swaddhiwudhipong^17^ | Thailand | 24 | BI, CI, HI | P, B, I, H, S | Chemical | Contemporary | No treatment |
| Therawiwat^18^ | Thailand | 12 | BI, CI, HI | I | None | Contemporary | No treatment |
| Toledo^19^ | Cuba | 12 | BI, CI, HI | P, B, I, T, H | Chemical | Contemporary | Some intervention |
| Vanlerberghe^20^ | Cuba | 13 | BI, HI | P, I, T, H | Chemical | Contemporary | Some intervention |
| Winch^21^ | Puerto Rico | 12 | BI, CI. HI | P, B, I, H, S | None | Contemporary | No treatment |

*Key* **BI**: Breteau index, **CI**: container index, **HI**: house index; **P**: print media, **B**: broadcast media, **I**: interactive media (lectures), **T**: training by staff, **H**: home educational visits, **S**: targeting of schoolchildren, **Rx**: treatment

^a^ Only interventions in control groups known for contemporary controls.

**Studies**

1. Armada Gessa J, Gonzalez R (1986) Application of environmental management principles in the program for eradication of *Aedes (Stegomyia) aegypti (Linneu, 1762)* in the Republic of Cuba, 1984. PAHO Bull 20: 186-193.
2. Avila Montes G, Martinez M, Sherman C, Cerna E (2004) Evaluacion de un modulo escolar sobre dengue y *Aedes aegypti* dirigido a escolares en Honduras. Pan Am J Public Health 16: 84-94.
3. Butraporn P, Saelim W, Sitapura P, Tantawiwat S (1999) Establishment of an environmental master team to control dengue haemorrhagic fever by local wisdom in Thailand. Dengue Bull 23: 99-104.
4. Eamchan P, Nisalak A, Foy H, Chareonsook O (1989) Epidemiology and control of dengue virus infections in Thai villages in 1987. Am J Trop Med Hyg 41: 8-326.
5. Espinoza-Gomez F, Hernandez-Suarez C, Coll-Cardenas R (2002) Educational campaign versus malathion spraying for the control of *Aedes aegypti* in Colima, Mexico. J Epidemiol Comm Health 56: 148-152.
6. Fernandez E, Leontsini E, Sherman C, Chan AST, Reyes CE, et al. (1998) Trial of a community-based intervention to decrease infestation of *Aedes aegypti* mosquitoes in cement washbasin in El Progreso, Honduras. Acta Trop 70: 171-183.
7. Jatanasen S (1967) Environmental manipulation and health education in Aedes aegypti control in Thailand. Bull World Health Org 36: 636-638.
8. Kay B, Nam V, Tien T, Thi Yen N, Vu Phong T, et al. (2002) Control of *Aedes* vectors of dengue in three provinces of Vietnam by use of *Mesocyclops (Copepoda)* and community-based methods validated by entomologic, clinical and serological surveillance. Am J Trop Med Hyg 66: 40-48.
9. Lardeux F, Sechan Y, Loncke S, Deparis X, Cheffort J, et al. (2002) Integrated control of peridomestic larval habitats of *Aedes* and *Culex* mosquitoes *(Diptera: Culicidae)* in atoll villages of French Polynesia. J Med Entomol 39: 493-498.
10. Leontsini E, Gil E, Kendall C, Clark G (1993) Effect of a community-based *Aedes aegypti* control programme on mosquito larval production sites in El Progreso, Honduras. Trans R Soc Trop Med Hyg 87: 267-271.
11. Lloyd L, Winch P, Ortega-Canto J, Kendall C (1992) Results of a community-based *Aedes aegypti* control program in Merida, Yucatan, Mexico. Am J Trop Med Hyg 46: 635-642.
12. Nam V, Yen N, Phong T, Ninh TU, Mai LQ, et al. (2005) Elimination of dengue by community programs using Mesocyclops (Copepoda) against Aedes aegypti in central Vietnam. Am J Trop Med Hyg 72: 67-73.
13. Phantumachinda B, Phanurai P, Samutrapongse W, Charoensook O (1985) Studies on community participation in *Aedes aegypti* control at Phanus Nikhom district, Chonburi Province, Thailand. Mosquito-Borne Dis Bull 2: 1-8.
14. Raju A (2003) Community mobilisation in *Aedes aegypti* control programme by source reduction in peri-urban district of Lautoka, Viti Levu, Fiji Islands. Dengue Bull 27: 149-155.
15. Sanchez L, Perez D, Perez T, Sosa T, Cruz, G. et al. (2005) Intersectoral coordination in *Aedes aegypti* control. A pilot project in Havana City, Cuba. Trop Med Int Health 10: 82-91.
16. Suroso H, Suroso T (1990) *Aedes aegypti*, control through source reduction by community efforts in Pekalongan, Indonesia. Mosquito-Borne Dis Bull 7: 59-62.
17. Swaddiwudhipong W, Chaovakiratipong C, Nguntra P, Koonchote S, Khumklam P, et al. (1992) Effect of health education on community participation in control of dengue haemorrhagic fever in an urban area of Thailand. Southeast Asian J Trop Med Public Health 23: 200-206.
18. Therawiwat M, Fungladda W, Kaewkungwai J, Imamee N, Steckler A (2005) Community-based approach for prevention and control of dengue haemorrhagic fever in Kanchanburi province, Thailand. Southeast Asian J Trop Med Public Health 36: 1439-1449.
19. Toledo M, Vanlerberghe V, Baly A, Ceballos E, Valdes, L. et al. (2007) Towards active community participation in dengue vector control: results from action research in Santiago de Cuba, Cuba. Trans R Soc Trop Med Hyg 101: 56-63.
20. Vanlerberghe V, Toledo M, Rodriguez M, Gomez D, Baly A, et al. (2009) Community involvement in dengue vector control: cluster randomised trial. Brit Med J 338: 1-7.
21. Winch P, Leontsini E, Rigau-Perez J, Ruiz-Pérez M, Clark GG, et al. (2002) Community-based dengue prevention programs in Puerto Rico: impact on knowledge, behaviour and residential mosquito infestation. Am J Trop Med Hyg 67: 363-370.
